# Associations of MTHFR gene polymorphism with lipid metabolism and risk of cerebral infarction in the Northwest Han Chinese population

**DOI:** 10.3389/fneur.2023.1152351

**Published:** 2023-03-31

**Authors:** Dong Guan, Yichun Ji, Xiaoyun Lu, Weiyi Feng, Wenbing Ma

**Affiliations:** ^1^Department of Pharmacology, The First Affiliated Hospital, Xi'an Jiaotong University, Xi'an, Shaanxi, China; ^2^Department of Hepatobiliary Surgery, The First Affiliated Hospital, Xi'an Jiaotong University, Xi'an, Shaanxi, China; ^3^Department of Biological Science and Bioengineering, Key Laboratory of Biomedical Information Engineering of the Ministry of Education, School of Life Science and Technology, Xi'an Jiaotong University, Xi'an, Shaanxi, China

**Keywords:** MTHFR, cerebral infarction, gene polymorphism, homocysteine, Northwest China

## Abstract

**Objective:**

Genetic variation in the methylenetetrahydrofolate reductase (MTHFR) gene may contribute to the development of cerebral infarction (CI); however, results have been inconsistent across studies with different populations, including studies of the Chinese population. The aim of this study was to analyze the effect of MTHFR gene polymorphism on serum lipid and homocysteine levels among patients with CI in the Northwest Chinese Han population.

**Patients and methods:**

A total of 521 CI patients and 524 non-CI controls were enrolled in the study. Polymerase chain reaction and hybridization were utilized to identify MTHFR gene polymorphisms. Multivariate logistic regression analysis was used to assess the associations of MTHFR gene polymorphism with risk of CI.

**Results:**

Frequencies of the TT genotype and the T allele were markedly higher among CI patients than among controls. After stratifying our data by sex and age, we determined that these differences in frequency of the TT genotype and the T allele were statistically significant among participants of two different age brackets and among men, but not among women (i.e., there were no statistically significant differences between female patients and female controls). CI patients and control participants with the CT or TT genotype had significantly higher homocysteine (Hcy) levels than those with the CC genotype. Among CI patients, CT/TT carriers showed significantly lower high-density lipoprotein cholesterol (HDL-C) and apolipoprotein A-I (ApoA-I) levels as compared with CC carriers, but there was no significant difference for control participants. Multivariable logistic regression analysis showed that drinking; smoking; diabetes mellitus; levels of Hcy, direct bilirubin (DB), indirect bilirubin (IB), ApoA-I, and total protein (TP); and TT genotype were significant independent risk factors for CI.

**Conclusions:**

The results suggested that the TT genotype of the MTHFR C677T gene polymorphism, which is associated with hyperhomocysteinemia (HHcy), might be of great clinical significance in the identification of new biomarkers for CI and in the development of individualized preventive and therapeutic strategies.

## 1. Introduction

The incidence of stroke has been increasing in recent years with improvements in people's living standards and aging of the population. The 2016 Global Burden of Disease study estimated that the overall lifetime risk of stroke among the Chinese population was 39.9%, which was the highest in the world (1). Cerebral infarction (CI) is the most common cerebrovascular disease, accounting for more than 70% of all strokes (2). Early identification of risk factors and effective preventive interventions are the keys to preventing CI.

Hyperhomocysteinemia (HHcy) and abnormal blood lipids have been widely accepted as major risk factors for CI (3). Hcy is an important metabolic intermediate that occurs during metabolism of methionine and cysteine. MTHFR is a key enzyme affecting the metabolism of Hcy; MTHFR catalyzes the reduction of 5,10-methylenetetrahydrofolate to 5-methyltetrahydrofolate and provides the methyl group for Hcy in methionine synthesis (4).

The C677T polymorphism of MTHFR alters nucleotide 677 by substituting cytosine for thymine. Three genotypes can therefore be determined for this polymorphism, namely, homozygous CC, homozygous TT, and heterozygous CT (5). The presence of MTHFR variants may affect MTHFR enzyme activity. Studies have shown that individuals with the homozygous variant TT have 70% lower enzyme activity, and heterozygous variant CT carriers have 35% lower enzyme activity, compared to the wild-type CC genotype (6). The frequency of the MTHFR C677T gene mutation varies among different ethnic groups (7). A high TT genotype frequency for the MTHFR gene, of approximately 25%, occurs in the Chinese population (8). MTHFR C677T polymorphism has been shown to decrease MTHFR activity and increase Hcy concentration, subsequently resulting in an increased risk of CI (9, 10). Hcy is normally present in the plasma in low concentration; however, when normal metabolism is disturbed, Hcy levels in the body are increased. An elevated Hcy level can lead to endothelial cell damage and dysfunction, promoting the expression of inflammatory factors, which in turn promotes platelet aggregation, resulting in damage to the blood vessels, which ultimately leads to an increased risk of CI (11). Although the MTHFR C677T polymorphism is recognized as a risk factor for CI, inconsistent results have been reported in studies of Thai, Tunisian, Turkish, Zambian, and Brazilian populations (12–14). In addition, the association between MTHFR gene polymorphism and serum lipid profiles still remains undetermined. The prevalence of MTHFR C677T polymorphism varies substantially in different regions of China and in other countries around the world (15, 16). Moreover, the association between MTHFR C677T polymorphism and the risk of developing CI in Northwestern China has not been studied to date. Therefore, the aim of the present study was to investigate the role of MTHFR C677T genotypes in risk of CI in the Northwest Chinese Han population.

## 2. Materials and methods

### 2.1. Participants

From June 2019 to June 2020, a total of 521 CI patients (163 women and 361 men) and 524 controls (159 women and 365 men) were recruited for participation from the First Affiliated Hospital of Xi'an Jiao Tong University, Shaanxi, China. Electrocardiography, cardiac and carotid ultrasonography, and magnetic resonance angiography (MRA) are performed routinely for cerebral infarction patients. In the present study, CI was diagnosed based on clinical findings, physical examination, brain CT scan, and/or magnetic resonance imaging (MRI). The exclusion criteria were as follows: chronic kidney disease, infectious diseases, cardiovascular disease, autoimmune disease, malignant tumor, relevant brain lesions detected on MRI, and use of lipid-lowering drugs at the time of the study. Gender- and age-matched unrelated healthy controls were selected from the general population at the same hospital during the same period. Healthy volunteers were required to meet the following criteria: no history of abnormal neurological examination results, immunological diseases, or stroke, and no cerebrovascular disease (i.e., negative imaging studies and no history of cerebrovascular disease). This study was approved by the Ethics Committee of the First Affiliated Hospital, of Xi'an Jiao Tong University, Shaanxi, China, and informed consent was obtained from all participants.

### 2.2. Analysis of lipids and other parameters

Anticoagulation peripheral blood samples were collected from all enrolled participants after an overnight fast. Sera were separated immediately and measured within 2 h. The parameters measured were TC, HDL, LDL-C, TG, ApoA-I, ApoB, ApoE, DB, IB, LP(a), and TP levels.

### 2.3. DNA extraction and genotyping

A 2-ml venous blood sample was drawn from the antecubital vein of each participant using a standard venipuncture technique with an ethylenediaminetetraacetic acid (EDTA) tube. Genomic DNA was extracted from whole blood using DNA isolation kits (JCQ-ENH Jinan Gospel Medical Technology Co., LTD) for peripheral blood and stored at −20°C. The 677C>T polymorphism of MTHFR was analyzed using the polymerase chain reaction (PCR) method. PCR analysis was performed according to the manufacturer's instructions: 50°C for 2 min, pre-denaturation at 95°C for 15 min, followed by 45 cycles at 94°C for 30 s, and 65°C for 45 s. PCR products were then reversely hybridized with gene chip technology. Finally, genotype was analyzed using a fluorescence detector (product model: Fascan 48S, production enterprise: Xi'an Tianlong Technology Co.).

### 2.4. Statistical analyses

SPSS statistical software, version 21.0 for Windows, was used for data analysis. Data are presented in the form of means and standard deviations (SDs) for continuous variables and in the form of numbers and percentages for categorical variables. Student's *t*-tests and chi-squared tests were initially used to identify significant differences between the CI and control groups. The genotype and allele frequencies of MTHFR C677T polymorphisms were tested for Hardy–Weinberg equilibrium and compared using the chi-squared test. A multivariate logistic regression analysis was performed to examine the contributions of MTHFR 677C>T alleles and other independent risk factors to the study outcomes. The independent variables used in the simple logistic regression model include drinking; smoking; hypertension; diabetes; levels of Hcy, TG, DB, IB, ApoA, ApoB, and TP; and TT genotype. A value of *p* < 0.05 was considered to indicate statistical significance.

## 3. Results

### 3.1. Baseline characteristics of the CI and control groups

The baseline clinical characteristics of the CI patients (*n* = 521) and healthy controls (*n* = 524) are summarized in Table 1. This study included a total of 1,045 individuals of Northwest Han Chinese ethnicity, comprising 361 male and 163 female CI patients (mean age = 54.07 years, SD =14.50), and 365 male and 159 female control participants (mean age = 53.36 years, SD = 14.67). The age and sex distributions of the two groups did not differ significantly (*p* = 0.431 and 0.789, respectively). There was no statistically significant difference in the presence of diabetes (*p* = 0.239) or hypertension (*p* = 0.234), but the proportion of participants reporting smoking and drinking was significantly higher in the case group than in the control group (*p* < 0.001 in both cases). CI patients had higher levels of Hcy concentration, triglycerides (TG), ApoA-I, ApoB, DB, IB, and TP compared with the control group; no significant differences were found between the two groups in levels of TC, high-density lipoprotein (HDL), LDL-C, ApoE, or lipoprotein(a) (Lp[a]) (all *p*s > 0.05).

**Table 1:** Characteristics of the study population.

**Factor**	**Control group (524)**	**CI group (521)**	***p*-value**
Age (years)	53.36 ± 14.67	54.07 ± 14.50	0.431
Sex (men/women)	365/159	361/163	0.789
Smoking	173 (33.02%)	260 (49.90%)	< 0.001
Drinking	90 (17.18%)	167 (47.08%)	< 0.001
Diabetes	290 (55.34%)	271 (52.02%)	0.239
Hypertension	75 (14.31%)	89 (17.08%)	0.234
Hcy	26.33 ± 16.49	29.19 ± 21.29	0.038
TC	3.70 ± 0.96	3.72 ± 0.96	0.737
HDL	0.97 ± 0.28	0.99 ± 0.27	0.402
LDL-C	2.18 ± 0.82	2.19 ± 0.84	0.914
TG	1.30 ± 0.90	1.50 ± 1.48	0.018
Cr	402.97 ± 83.00	407.76 ± 119.60	< 0.001
ApoA-I	1.06 ± 0.21	1.10 ± 0.20	0.002
ApoB	0.71 ± 0.21	0.74 ± 0.21	0.017
ApoE	35.33 ± 17.31	35.66 ± 15.60	0.766
DB	3.43 ± 2.73	4.30 ± 2.95	< 0.001
IB	7.48 ± 5.49	9.74 ± 6.03	< 0.002
LP(a)	322.71 ± 288.98	312.77 ± 301.73	0.617
TP	63.11 ± 7.16	64.77 ± 5.86	< 0.001

To further explore the effects of potential confounding factors on lipid fluctuations, we performed analyses stratified by age (dichotomized into ≤ 60 and >60 years) and sex (Table 2). Among the younger group (age ≤ 60 years), serum ApoA-I, IB, DB, and total protein levels were all significantly higher among patients with CI than among the control participants (all *p*s < 0.001); among the older group (age > 60 years), TG and Hcy levels were significantly higher among patients with CI than among the controls (both *p*s < 0.05). Among men, Hcy, TG, ApoA-I, ApoB, IB, DB, and TP levels were significantly higher in patients with CI than in controls; among women, ApoA-I, IB, and TP levels were significantly higher in CI patients than in controls.

**Table 2:** Stratified analyses of lipid levels by age and sex.

**Factors**	**Age** ≤ **60**			**Age** > **60**			**Men**			**Women**		
	**CI group**	**Control group**	***p***	**CI group**	**Control group**	***p***	**CI group**	**Control group**	***P***	**CI group**	**Control group**	***p***
Hcy	27.86 ± 20.34	26.38 ± 16.99	0.364	31.63 ± 22.88	26.47 ± 15.27	0.039	33.25 ± 22.75	28.10 ± 17.34	0.003	18.37 ± 11.26	22.41 ± 13.64	0.220
TC	3.68 ± 0.93	3.68 ± 0.95	0.996	3.79 ± 1.03	3.74 ± 0.99	0.633	3.65 ± 1.03	3.60 ± 0.91	0.473	3.88 ± 0.75	3.93 ± 1.04	0.608
HDL	0.98 ± 0.28	0.95 ± 0.28	0.479	1.01 ± 0.26	1.02 ± 0.28	0.924	0.94 ± 0.25	0.92 ± 0.25	0.347	1.11 ± 0.29	1.10 ± 0.30	0.625
LDL-C	2.14 ± 0.75	2.19 ± 0.80	0.479	2.27 ± 0.99	2.17 ± 0.86	0.369	2.15 ± 0.88	2.13 ± 0.77	0.799	2.27 ± 0.71	2.29 ± 0.92	0.830
TG	1.58 ± 1.74	1.37 ± 0.98	0.06	1.32 ± 0.69	1.13 ± 0.63	0.017	1.52 ± 1.64	1.31 ± 0.91	0.040	1.42 ± 0.99	1.29 ± 0.89	0.251
ApoA	1.09 ± 0.20	1.05 ± 0.21	0.011	1.12 ± 0.21	1.08 ± 0.22	0.131	1.06 ± 0.19	1.03 ± 0.19	0.017	1.21 ± 0.20	1.14 ± 0.23	0.019
ApoB	0.74 ± 0.20	0.70 ± 0.21	0.065	0.75 ± 0.23	0.72 ± 0.21	0.171	0.74 ± 0.22	0.70 ± 0.20	0.031	0.74 ± 0.19	0.72 ± 0.23	0.336
ApoE	35.36 ± 13.54	36.07 ± 18.71	0.597	35.14 ± 15.01	33.62 ± 13.40	0.372	33.71 ± 11.86	33.70 ± 17.08	0.991	39.34 ± 17.94	39.15 ± 17.27	0.930
DB	4.23 ± 3.00	3.22 ± 2.89	< 0.001	4.42 ± 2.83	3.97 ± 2.25	0.120	4.59 ± 3.20	3.55 ± 2.96	< 0.001	3.56 ± 2.07	3.19 ± 2.13	0.131
IB	9.76 ± 6.22	6.66 ± 4.96	< 0.001	9.59 ± 5.40	9.55 ± 6.37	0.959	10.01 ± 6.18	7.68 ± 5.93	< 0.001	8.97 ± 5.38	7.16 ± 4.65	0.002
Lp(a)	306.92 ± 292.02	324.01 ± 270.76	0.457	323.42 ± 321.36	320.66 ± 327.05	0.943	310.39 ± 298.60	317.86 ± 290.32	0.750	317.90 ± 311.67	334.95 ± 285.66	0.648
TP	65.01 ± 5.84	62.94 ± 7.70	< 0.001	64.26 ± 5.90	63.49 ± 5.64	0.244	64.05 ± 5.97	62.64 ± 7.08	0.005	64.48 ± 5.23	64.16 ± 7.22	0.001

### 3.2. Association of MTHFRC677T with risk of CI

The genotype distributions of the MTHFR C677T gene polymorphism were in Hardy–Weinberg equilibrium in both patients and control subjects (*p* = 0.209 and 0.759, respectively), suggesting that the selected samples were representative (Table 3). The CT genotype (at 44.53 and 50.57% in the CI group and the control group, respectively) was the most common in both groups, followed by the TT (39.73 and 26.91%) and CC (15.74 and 22.52%) genotypes. The allele frequencies of C and T polymorphisms were 38.00 and 62.00% among the CI group and 47.81 and 52.19% among the control participants, respectively. There was a statistically significant difference between the CI group and the control group in terms of the frequencies of MTHFR C677T genotypes (χ^2^ = 21.18, *p* < 0.001) and alleles (χ^2^ =20.49, *p* < 0.001). The odds ratio (OR) for the CI group was 1.791 (95% CI = 1.379–2.324, *p* < 0.001) for the TT genotype and 0.643 (95% CI = 0.470–0.848, *p* < 0.001) for the CC genotype. The frequency of the T allele was significantly higher in CI patients than in control participants (OR = 1.494, 95% CI = 1.126–1.778, *p* < 0.001), and the frequency of the C allele was significantly lower (OR = 0.669, 95% CI = 0.562–0.797, *p* < 0.001). The results suggested that the TT genotype and T allele were significantly associated with a higher risk of CI compared with the risk among those carrying the CC genotype and C allele.

**Table 3:** The distributions of genotypes and alleles of the MTHFR C677T gene among CI patients and controls.

**Genotype, *n***	**CI group (*n* = 517)**	**Control group (*n* = 517)**	**OR (95% CI)**	***p^*a*^***	**χ^2^**	***p* ^b^**
CC	82	118	0.643 (0.470, 0.878)	0.005	21.18	< 0.001
CT	232	265	0.785 (0.615, 1.001)	0.050		
TT	207	141	1.791 (1.379, 2.324)	< 0.001		
*HWE*	χ^2^ = 0.094, *p* = 0.759	χ^2^ = 3.33, *p* = 0.50				
**Alleles**, ***n***						
C	396	501	0.669 (0.562, 0.797)	< 0.001	20.489	< 0.001
T	646	547	1.494 (1.1255, 1.778)	< 0.001		

CI, cerebral infarction; HWE, Hardy–Weinberg equilibrium.

^a^*p* and OR (95% CI) values were calculated by logistic regression adjusted for age, gender, hypertension, diabetes, smoking, and drinking.

^b^*p*-values were calculated from two-sided chi-squared tests or Fisher's exact tests.

To further determine the relationship between the MTHFR C677T genotype and CI, we stratified the participants based on age (dichotomized into ≤ 60 and >60 years) and sex. The results showed that the frequencies of the TT genotype and T allele were significantly higher in the CI group compared with the control group among both age groups (age ≤ 60 years: TT genotype OR = 1.706, 95% CI = 1.246–2.336, *p* < 0.001; T allele OR =1.398, 95% CI = 1.137–1.720, *p* = 0.002; age > 60 years: TT genotype OR =1.980, 95% CI = 1.238–3.168, *p* = 0.004; T allele OR = 1.746, 95% CI = 1.265–2.410, *p* = 0.001). This was also the case among men (TT genotype OR = 1.971, 95% CI = 1.445–2.689, *p* < 0.001; T allele OR =1.638, 95% CI = 1.328–2.022, *p* < 0.001), but not among women (TT genotype OR = 1.426, 95% CI = 0.878–2.314, *p* = 0.151; T allele OR = 1.223, 95% CI = 0.595–1.671, *p* = 0.206) (Table 4).

**Table 4:** Stratified analyses of the association between MTHFR C677T polymorphism and risk of CI.

**Genotype, *n***	**Age** ≤ **60**				**Age** > **60**				**Men**				**Women**			
	**CI**	**Control**	**OR (95% CI)**	***p***	**CI**	**Control**	**OR (95% CI)**	***p***	**CI**	**Control**	**OR (95% CI)**	***p***	**CI**	**Control**	**OR (95% CI)**	***p***
	***n*** = **362**	***n*** = **362**			***n*** = **159**	***n*** = **152**			***n*** = **361**	***n*** = **365**			***n*** = **160**	***n*** = **159**		
CC	59	79	0.722 (0.497, 1.049)	0.087	23	39	0.490 (0.276, 0.869)	0.014	51	83	0.559 (0.381, 0.821)	0.003	31	35	0.851 (0.495, 1.465)	0.561
CT	167	196	0.769 (0.575, 1.028)	0.076	65	69	0.832 (0.531, 1.304)	0.422	156	182	0.765 (0.571, 1.025)	0.073	76	83	0.828 (0.534, 1.286)	0.401
TT	136	97	1.706 (1.246,2.336)	0.001	71	44	1.980 (1.238, 3.168)	0.004	154	100	1.971 (1.445, 2.689)	< 0.001	53	41	1.426 (0.878, 2.314)	0.151
Alleles, *n*																
C	285	354	0.715 (0.581, 0.880)	0.002	111	147	0.573 (0.415, 0.790)	0.001	258	348	0.610 (0.495, 0.753)	< 0.001	138	153	0.818 (0.599, 1.117)	0.206
T	439	390	1.398 (1.137, 1.720)	0.002	207	157	1.746 (1.265, 2.410)	0.001	464	382	1.638 (1.328, 2.022)	< 0.001	182	165	1.223 (0.595, 1.671)	0.206

### 3.3. Relationships between serum lipid profile and MTHFR C677T alleles

Table 5 presents the association between serum lipid profiles and MTHFR C677T polymorphisms. Our data showed that CI patients and control participants who were CT/TT carriers had significantly higher Hcy levels than those who were CC carriers (both *p*s < 0.001). Among CI patients, CT/TT carriers showed significantly lower HDL-C and ApoA-I levels as compared with CC carriers, but there was no significant difference for control participants. Additionally, no significant differences between the two genotypes were observed in other blood lipid levels for CI patients or controls.

**Table 5:** Relationships between serum lipid profiles and MTHFR C677T polymorphism among CI patients and control participants.

**Factor**	**CI**			**Control**		
	**CC**	**CT/TT**	***p***	**CC**	**CT/TT**	***p***
Hcy	20.52 ± 13.51	30.78 ± 20.07	< 0.001	22.55 ± 12.04	27.49 ± 15.22	0.027
TC	3.75 ± 0.79	3.71 ± 0.99	0.792	3.66 ± 1.07	3.71 ± 0.93	0.661
HDL	1.04 ± 0.27	0.98 ± 0.26	0.044	0.99 ± 0.34	0.97 ± 0.26	0.482
LDL-C	2.18 ± 0.68	2.19 ± 0.86	0.954	2.12 ± 0.90	2.20 ± 0.80	0.394
TG	1.37 ± 0.74	1.52 ± 1.51	0.375	1.33 ± 0.86	1.30 ± 0.91	0.768
ApoA	1.15 ± 0.19	1.09 ± 0.20	0.030	1.06 ± 0.26	1.06 ± 0.20	0.535
ApoB	0.74 ± 0.19	074 ± 0.22	0.922	0.67 ± 0.22	072 ± 0.21	0.08
ApoE	34.58 ± 12.50	35.85 ± 16.09	0.52	37.64 ± 22.70	34.67 ± 15.43	0.145
DB	3.87 ± 2.81	4.38 ± 2.97	0.159	3.52 ± 2.83	3.41 ± 2.71	0.713
IB	9.03 ± 4.26	9.88 ± 6.30	0.251	7.26 ± 5.69	7.54 ± 5.44	0.634
Lp(a)	254.57 ± 240.53	323.68 ± 310.92	0.071	323.73 ± 277.44	322.41 ± 292.57	0.969
TP	65.31 ± 6.50	64.66 ± 5.73	0.368	62.83 ± 6.96	63.18 ± 7.22	0.640

To investigate potential sex-specific effects, we further performed stratified analyses according to sex. Among male CI patients, CT/TT carriers had significantly higher Hcy levels than CC carriers (*p* < 0.05); this trend was also observed in female CI patients and in male and female controls, but in these groups, the difference did not reach statistical significance. Among female CI patients, CC carriers had significantly higher HDL-C levels than CT/TT carriers. Finally, among male control patients CT/TT carriers had higher ApoB levels and lower ApoE levels compared to CC carriers (Table 6).

**Table 6:** Stratified analyses of MTHFR C677T polymorphism and serum lipid profiles.

**Factor**	**CI**						**Control**					
	**Men**			**Women**			**Men**			**Women**		
	**CC**	**CT/TT**	***p***	**CC**	**CT/TT**	***p***	**CC**	**CC/TT**	***p***	**CC**	**CC/TT**	***p***
Hcy	22.74 ± 15.52	35.11 ± 23.30	< 0.001	15.80 ± 5.41	18.94 ± 12.13	0.227	24.21 ± 12.66	29.05 ± 18.30	0.094	19.490 ± 10.36	23.53 ± 14.62	0.210
TC	3.55 ± 0.80	3.67 ± 1.07	0.443	4.09 ± 0.66	3.83 ± 0.76	0.091	3.54 ± 0.94	3.61 ± 0.90	0.597	3.95 ± 1.31	3.93 ± 0.96	0.940
HDL	0.96 ± 0.20	0.94 ± 0.25	0.627	1.18 ± 0.29	1.07 ± 0.26	0.046	0.94 ± 0.30	0.92 ± 0.24	0.565	1.11 ± 0.39	1.09 ± 0.27	0.837
LDL-C	2.10 ± 0.67	2.16 ± 0.91	0.656	2.34 ± 0.69	2.25 ± 0.72	0.595	2.02 ± 0.76	2.16 ± 0.77	0.181	2.32 ± 1.13	2.28 ± 0.85	0.826
TG	1.37 ± 0.60	1.55 ± 1.70	0.457	1.36 ± 0.87	1.44 ± 0.92	0.654	1.36 ± 0.95	1.30 ± 0.90	0.588	1.24 ± 0.63	1.30 ± 0.95	0.761
ApoA	1.09 ± 0.15	1.06 ± 0.19	0.319	1.27 ± 0.20	1.19 ± 0.20	0.064	1.03 ± 0.20	1.02 ± 0.19	0.665	1.12 ± 0.34	1.15 ± 0.19	0.620
ApoB	0.73 ± 0.19	0.74 ± 0.22	0.617	0.76 ± 0.18	0.74 ± 0.20	0.524	0.66 ± 0.19	0.72 ± 0.20	0.033	0.71 ± 0.27	0.72 ± 0.21	0.897
ApoE	31.53 ± 9.19	33.96 ± 12.54	0.190	40.95 ± 15.91	40.96 ± 22.45	0.999	37.63 ± 26.04	32.63 ± 13.60	0.040	37.67 ± 13.62	39.61 ± 18.30	0.594
DB	4.16 ± 3.32	4.68 ± 3.18	0.288	3.36 ± 1.51	3.61 ± 2.19	0.567	3.59 ± 3.02	3.53 ± 2.94	0.869	3.34 ± 2.31	3.15 ± 2.09	0.655
IB	8.71 ± 4.18	10.30 ± 6.52	0.096	9.58 ± 4.40	8.82 ± 5.60	0.494	7.55 ± 6.29	7.63 ± 5.68	0.908	6.53 ± 3.75	7.33 ± 4.86	0.393
Lp(a)	247.68 ± 246.92	321.74 ± 305.32	0.105	268.92 ± 321.10	328.89 ± 326.92	0.396	307.41 ± 267.76	320.16 ± 297.14	0.758	358.00 ± 298.52	327.82 ± 282.78	0.615
TP	64.22 ± 7.24	64.01 ± 5.74	0.823	67.20 ± 4.48	8.82 ± 5.60	0.410	62.45 ± 7.38	7.64 ± 5.68	0.782	63.74 ± 5.82	64.27 ± 7.58	0.715

### 3.4. Logistic regression analysis of CI risk factors

Univariate analysis showed that the traditional CI risk factors (smoking; drinking; and levels of TG, ApoA-I, ApoB, DB, IB, and total protein) were significantly associated with the presence of CI (all *p*s < 0.05). Furthermore, multivariate logistic regression analysis was performed to analyze the independent risk factors for CI; this analysis identified drinking (OR = 1.718, 95% CI = 1.179–2.503, *p* = 0.005); smoking (OR = 1.724, 95% CI = 1.239–2.399, *p* = 0.001); diabetes mellitus (OR = 1.504, 95% CI = 1.020–2.219, *p* = 0.04); Hcy (OR = 1.010, 95% CI = 1.001–1.018, *p* = 0.28), DB (OR = 1.086, 95% CI = 1.013–1.164, *p* = 0.021), IB (OR = 1.035, 95% CI = 1.002–1.070, *p* = 0.036), ApoA-I (OR = 4.239, 95% CI = 1.893–9.492, *p* < 0.001), and TP (OR = 1.025, 95% CI = 1.000–1.051, *p* = 0.048); and TT genotype (OR = 1.778, 95% CI = 1.218–2.596; *p* = 0.003) as significant independent risk factors for CI (Table 7).

**Table 7:** Logistic regression analysis of risk of CI in the population of Northwest China.

**Variable**	***p*-value**	**OR (95% CI)**
Drinking	0.005	1.718 (1.179–2.503)
Smoking	0.001	1.724 (1.239–2.399)
Hypertension	0.659	0.937 (0.702–1.251)
Diabetes	0.040	1.504 (1.020–2.219)
Hcy	0.028	1.010 (1.001–1.018)
TG	0.136	1.151 (0.957–1.385)
DB	0.210	1.086 (1.013–1.164)
IB	0.036	1.035 (1.002–1.070)
ApoA	0.000	4.239 (1.893–9.492)
ApoB	0.430	1.353 (0.638–2.869)
Total protein	0.048	1.025 (1.000–1.051)
TT	0.003	1.778 (1.218–2.596)

In subgroup analyses stratified by age and sex, the results suggested that the MTHFR TT genotype was associated with a significantly increased risk of CI among participants in both age brackets (age ≤ 60 years: OR = 1.706, 95% CI = 1.246–2.336, *p* = 0.001; age > 60 years: OR = 2.437, 95% CI = 1.264–4.700, *p* = 0.008) and of both sexes (men: OR = 1.591, 95% CI = 1.023–2.474, *p* = 0.039; women: OR = 2.506, 95% CI = 1.162–5.406, *p* = 0.019) (Table 8).

**Table 8:** Multiple logistic regression analysis for CI patients and control participants.

**TT**		
	**OR (95% CI)**	***p*** **-value**
Age ≤ 60	1.706 (1.246–2.336)	0.001
Age > 60	2.437 (1.264–4.700)	0.008
Men	1.591 (1.023–2.474)	0.039
Women	2.506 (1.162–5.406)	0.019

## 4. Discussion

In China, the stroke burden is expected to increase dramatically due to the growing and aging population and a trend toward increasing prevalence of risk factors (17). Despite advances in the prevention and treatment of CI, there are still few therapies that have been shown to improve outcomes after CI (18). Therefore, active investigation of the pathogenesis of CI and preventive measures against CI are of great importance. This study analyzed the relationship between genetic polymorphisms of MTHFR and CI in the Han Chinese population in Northwest China.

Numerous studies have shown that various environmental variables and genetic variations contribute to the etiology of CI (19). Previously published studies have demonstrated associations between MTHFR polymorphisms and CI, although the results have been mixed (20, 21).

The frequency of the MTHFR C677T gene mutation varies among different ethnic groups (7). The frequency of the T allele has often been reported to be high among Europeans and North Americans (with a gradient of increasing frequency from north to south) and low among East Asians and Africans; however, the opposite trend in terms of geographical gradient has been reported among Chinese populations (14, 15). There is a high frequency for the TT genotype of the MTHFR gene, of ~25%, in the Chinese population (8). In the present study, genotype CT (44.5%) was the predominant genotype among patients with CI, followed by TT (39.7%) and CC (15.7%); the corresponding frequencies among the control participants were 50.6, 26.9, and 22.5%, respectively. The T allele was the most common, occurring at frequencies of 62.0 and 52.2% among CI patients and control participants, respectively. Our results indicated that there are significant differences in the distribution of frequencies of genotypes and alleles relating to MTHFR C677T polymorphism between the Northwest Chinese Han population and the Hakka population in China (14), which also suggests that the distribution of MTHFR C677T gene polymorphisms may vary among ethnic groups and geographic regions. We also found that the TT genotype and T allele increased CI risk by 1.791- and 1.494-fold, respectively, overall. This suggests that the MTHFR TT genotype and T allele are associated with susceptibility to CI in the Northwest Chinese Han population. These results are consistent with those of previous studies (22).

MTHFR variants may affect MTHFR enzyme activity. Studies have shown that individuals with the homozygous variant (TT) have 70% lower enzyme activity and those with the heterozygous variant (CT) have 35% lower enzyme activity compared to the wild-type (CC) genotype (6). The TT genotype is commonly linked with elevated Hcy levels through reduction in the activity of MTHFR (23). In the present study, Hcy level was significantly correlated with genotype (CT/TT) of the MTHFR C677T gene polymorphism among both the control and the CI groups, which was consistent with findings in Asian, Singaporean, Malaysian, Tunisian, Chinese, and Polish populations (24–28). However, studies of Indonesian, Zambian, and Brazilian populations have found no association between MTHFR C677T polymorphism and risk of CI (29–31). After stratification by sex, no significant difference in Hcy levels was observed between CT/TT carriers and CC carriers among female CI patients. These inconsistent results might be the result of variations in participant ethnicities, detection techniques, sample sizes, research designs, and other factors. Hcy is a sulfur-containing amino acid generated by methionine metabolism; it can damage vascular endothelial cells; promote vascular smooth muscle cell proliferation, platelet aggregation, and thrombosis; and enhance coagulation (32). HHcy has been proven to be associated with cerebrovascular disease and cardiovascular disorders (33, 34). Our results showed that serum Hcy levels were significantly higher in patients with CI than in the control group, suggesting that MTHFR gene polymorphism could be an underlying genetic risk factor for HHcy.

It is well-known that many traditional risk factors, such as hypertension, smoking, drinking, and dyslipidemia, are strongly associated with increased risk of CI (35, 36). In the present study, multivariate logistic regression analysis showed that drinking, smoking, diabetes mellitus, and elevated levels of DB, IB, ApoA-I, and TP were significant independent risk factors for CI. Dyslipidemia is affected by genetic and environmental factors and by the interaction between them (37). At present, the correlation between MTHFR gene polymorphism and serum lipid profiles is still controversial in diverse ethnic groups. A previous study demonstrated that dyslipidemia caused by MTHFR C667T gene polymorphism plays a major role in prediction of ischemic stroke (38, 39). It has also been reported that, when MTHFR C677T polymorphism is detected in renal transplant patients, the C allele has a protective effect on blood lipid concentration, while the T allele has an unfavorable effect on blood lipid concentration (40). In the present study, participants with the T allele had significantly higher TC and LDL-C levels compared to those with the CC genotype. Zhang et al. showed that MTHFR 677T carriers exhibit significantly increased serum levels of TC and LDL-C (41). However, we note that not all studies have observed a significant association between these two polymorphisms and serum lipid levels. In an interesting study by Chen et al., the authors showed that MTHFR 677T carriers had significantly lower TC, LDL-C, folate, and vitamin B12 levels than those with the CC genotype (42). Another Chinese clinical study found that plasma Hcy levels and MTHFR C677T polymorphism were not related to serum lipid levels (43). These differences in findings may be related to differences in sample size and ethnicity. In the present study, among both CI patients and control participants, no significant difference between CC and CT/TT carriers was observed in TC, LDL-C, or TG levels. Previous studies have found that HHcy increases serum levels of TC and TG (44) and reduces serum levels of ApoA1 and HDL-C (45). Low HDL-C levels may increase the risk of CI by 1.8-fold and increase the risk of stroke by 1.5-fold in the Chinese population (46). Our previous research have shown that ApoA-I is a major structural and functional protein component of HDL, which plays an essential role in coronary artery disease (47). Ljunggren et al. found that low HDL-C and ApoA-I levels are associated with an increased risk of cerebrovascular disease (48). Our results showed that, among CI patients, T allele carriers had lower levels of HDL-C and ApoA-I than non-carriers of the T allele. After stratification by sex, we found that, among female CI patients, CC carriers had significantly higher HDL-C levels than CT/TT carriers.

The primary strength of this study is that this is the first study investigating the relationship between CI and MTHFR gene polymorphism in the Northwest Han Chinese population. Investigation of the association of lipid levels with MTHFR gene polymorphisms was included in the final analysis, and any potential confounding variables or comorbidities were eliminated. There were some inherent limitations to our study: (1) due to a lack of original data and the size of this retrospective investigation, it was difficult to examine potential gene–environment interactions; (2) the study's limited sample size might partially contribute to the volatility of the results; and (3) the study was conducted only in the Northwest Chinese population, and further investigation is needed to determine whether these findings will also hold in other populations.

## 5. Conclusions

This study suggests that the TT genotype of MTHFR is associated with CI in the Northwest Han Chinese population. Drinking; smoking; diabetes mellitus; elevated levels of Hcy, DB, IB, ApoA-I, and TP; and the TT genotype are independent risk factors for CI among patients in Northwest China. MTHFR gene polymorphism was found to be associated with CI, with this association being partly mediated by blood lipids and potentially also being mediated by non-lipid pathways. Due to the small sample size, more research with a larger sample is required to support our findings. Our research provides useful genomic data which may assist in optimizing individual preventative and therapeutic strategies.

## Data availability statement

The original contributions presented in the study are included in the article/supplementary material, further inquiries can be directed to the corresponding author.

## Ethics statement

The studies involving human participants were reviewed and approved by the Ethical Committee of the First Affiliated Hospital of Xi'an Jiao Tong University. The patients/participants provided their written informed consent to participate in this study.

## Author contributions

WM and DG conceived and designed the experiments. WM and WF contributed to the writing of the manuscript. DG and YJ recruited the study participants and collected clinical data. DG and XL helped to analyze the data. All authors have read and approved the final manuscript.

## References

[1] Li A-l Zhu S Hu Z-h Peng Q Fang X Zhang Y-y. The distribution and epidemic characteristics of cerebrovascular disease in followed-up hypertension patients. Sci Rep. (2021) 11:9366. 10.1038/s41598-021-88127-5 33931694 PMC8087808

[2] He J Xuan X Jiang M Li J Li N Nie T. Long non-coding RNA SNHG1 relieves microglia activation by downregulating miR-329-3p expression in an in vitro model of cerebral infarction. Exp Ther Med. (2021) 22:1148. 10.3892/etm.2021.10581 34504593 PMC8393422

[3] Hao L Chen L Sai X Liu Z Yang G Yan R. Synergistic effects of elevated homocysteine level and abnormal blood lipids on the onset of stroke. Neural Regener Res. (2013) 8:2923–31. 10.3969/j.issn.1673-5374.2013.31.005 25206613 PMC4146172

[4] Taiwo TE Cao X Cabrera RM Lei Y Finnell RH. Approaches to studying the genomic architecture of complex birth defects. Prenat Diagn. (2020) 40:1047–55. 10.1002/pd.5760 32468575 PMC8117178

[5] Zhang S Wang T Wang H Tang J Hou A Yan X. Effects of individualized administration of folic acid on prothrombotic state and vascular endothelial function with H-type hypertension A double-blinded, randomized clinical cohort study. Medicine. (2022) 101:e28628. 10.1097/MD.0000000000028628 35060542 PMC8772678

[6] Yang S Lee J Park Y Lee EK Hwangbo Y Ryu J. Interaction between alcohol consumption and methylenetetrahydrofolate reductase polymorphisms in thyroid cancer risk: National Cancer Center cohort in Korea. Sci Rep. (2018) 8:4077. 10.1038/s41598-018-22189-w 29511243 PMC5840348

[7] Wang X Fu J Li Q Zeng D. Geographical and ethnic distributions of the MTHFR C677T, A1298C and MTRR A66G Gene Polymorphisms in Chinese Populations: a meta-analysis. PLoS ONE. (2016) 11:0152414. 10.1371/journal.pone.0152414 27089387 PMC4835080

[8] Zhang J Li J Chen S Gao L Yan X Zhang M. Modification of platelet count on the association between homocysteine and blood pressure: a moderation analysis in chinese hypertensive patients. Int J Hyperten. (2020) 2020:5983574. 10.1155/2020/5983574 32128262 PMC7048938

[9] Chang H-L Chen G-R Hsiao P-J Chiu C-C Tai M-C Kao C-C. Decisive evidence corroborates a null relationship between MTHFR C677T and chronic kidney disease A case-control study and a meta-analysis. Medicine. (2020) 99:e21045. 10.1097/MD.0000000000021045 32702845 PMC7373545

[10] Jankovic M Petrovic B Novakovic I Brankovic S Radosavljevic N Nikolic D. The genetic basis of strokes in pediatric populations and insight into new therapeutic options. Int J Mol Sci. (2022) 23:1601. 10.3390/ijms23031601 35163523 PMC8835808

[11] Li H Zhang P Yuan S Tian H Tian D Liu M. Modeling analysis of the relationship between atherosclerosis and related inflammatory factors. Saudi J Biol Sci. (2017) 24:1803–9. 10.1016/j.sjbs.2017.11.016 29551927 PMC5851939

[12] Hou J Zeng X Xie Y Wu H Zhao P. Genetic polymorphisms of methylenetetrahydrofolate reductase C677T and risk of ischemic stroke in a southern Chinese Hakka population. Medicine. (2018) 97. 10.1097/MD.0000000000013645 30572478 PMC6320192

[13] Chutinet A Suwanwela NC Snabboon T Chaisinanunkul N Furie KL Phanthumchinda K. Association between genetic polymorphisms and sites of cervicocerebral artery atherosclerosis. J Stroke Cerebrovasc Dis. (2012) 21:379–85. 10.1016/j.jstrokecerebrovasdis.2010.10.002 21296594

[14] HouJ Zeng X Xie Y Wu H Zhao P. Genetic polymorphisms of methylenetetrahydrofolate reductase C677T and risk of ischemic stroke in a southern Chinese Hakka population. Medicine. (2018) 97:e13645. 30572478 10.1097/MD.0000000000013645 PMC6320192

[15] Yang B Liu Y Li Y Fan S Zhi X Lu X. Geographical distribution of MTHFR C677T, A1298C and MTRR A66G gene polymorphisms in China: findings from 15357 adults of Han nationality. PLoS ONE. (2013) 8:e57917. 10.1371/journal.pone.0057917 23472119 PMC3589470

[16] Nefic H Mackic-Djurovic M Eminovic I. The frequency of the 677C>T and 1298A>C polymorphisms in the methylenetetrahydrofolate reductase (MTHFR) gene in the population. Med Arch. (2018) 72:164–9. 10.5455/medarh.2018.72.164-169 30061759 PMC6021155

[17] Li S Chen L Xu C Qu X Qin Z Gao J. Expression profile and bioinformatics analysis of circular RNAs in acute ischemic stroke in a South Chinese Han population. Sci Rep. (2020) 10:10138. 10.1038/s41598-020-66990-y 32576868 PMC7311391

[18] Owen B Akbik O Torbey M Davis H Carlson AP. Incidence and outcomes of intracerebral haemorrhage with mechanical compression hydrocephalus. Stroke Vasc Neurol. (2021) 6:328–36. 10.1136/svn-2020-000401 33419863 PMC8485232

[19] Zhang L-J Yuan B Li H-H Tao S-B Yan H-Q Chang L. Associations of genetic polymorphisms of SAA1 with cerebral infarction. Lipids Health Dis. (2013) 12:130. 10.1186/1476-511X-12-130 23987125 PMC3765816

[20] Huang LW Li LL Li J Chen XR Yu M. Association of the methylenetetrahydrofolate reductase (MTHFR) gene variant C677T with serum homocysteine levels and the severity of ischaemic stroke: a case-control study in the southwest of China. J Int Med Res. (2022) 50:3000605221081632. 10.1177/03000605221081632 35225709 PMC8894968

[21] Hashemi SM Ramroodi N Amiri Fard H Talebian S Haghighi Rohani M Rezaei M. Common variations in prothrombotic genes and susceptibility to ischemic stroke in young patients: a case-control study in southeast Iran. Medicina. (2019) 55:47. 10.3390/medicina55020047 30781868 PMC6409550

[22] Arina CA Amir D Siregar Y Sembiring RJ. The role of polymorphism gen methylene tetra hydrofolate reductase (MTHFR) C677T in ischaemic stroke patients with and without hypertension. Open Access Maced J Med Sci. (2019) 7:29–32. 10.3889/oamjms.2019.026 30740155 PMC6352466

[23] Lisboa JVC Ribeiro MR Luna RCP Lima RPA Nascimento R Monteiro M. Food intervention with folate reduces TNF-α and interleukin levels in overweight and obese women with the MTHFR C677T polymorphism: a randomized trial. Nutrients. (2020) 12:361. 10.3390/nu12020361 32019154 PMC7071147

[24] Moe KT Woon FP De Silva DA Wong P Koh TH Kingwell B. Association of acute ischemic stroke with the MTHFR C677T polymorphism but not with NOS3 gene polymorphisms in a Singapore population. Eur J Neurol. (2008) 15:1309–14. 10.1111/j.1468-1331.2008.02308.x 19049547

[25] Mejia Mohamed EH Tan KS Ali JM Mohamed Z. TT genotype of the methylenetetrahydrofolate reductase C677T polymorphism is an important determinant for homocysteine levels in multi-ethnic Malaysian ischaemic stroke patients. Ann Acad Med Singap. (2011) 40:186–91. 10.47102/annals-acadmedsg.V40N4p186 21678004

[26] Fekih-Mrissa N Mrad M Klai S Mansour M Nsiri B Gritli N. Methylenetetrahydrofolate reductase (C677T and A1298C) polymorphisms, hyperhomocysteinemia, and ischemic stroke in Tunisian patients. J Stroke Cerebrovasc Dis. (2013) 22:465–9. 10.1016/j.jstrokecerebrovasdis.2013.03.011 23642756

[27] Zhang W Wang Y Bi G. Quantitative assessment of association between rs1801133 polymorphism and susceptibility to stroke. Cell Biochem Biophys. (2015) 71:85–98. 10.1007/s12013-014-0166-3 25107455

[28] Kumar P Mishra A Prasad MK Verma V Kumar A. Relationship of methylenetetrahydrofolate reductase (MTHFR) C677T variation with susceptibility of patients with ischemic stroke: a meta-analysis. Cureus. (2022) 14:e28218. 10.7759/cureus.28218 36017481 PMC9393322

[29] Atadzhanov M Mwaba MH Mukomena PN Lakhi S Rayaprolu S Ross OA. Association of the APOE, MTHFR and ACE genes polymorphisms and stroke in Zambian patients. Neurol Int. (2013) 5:e20. 10.4081/ni.2013.e20 24416484 PMC3883065

[30] Marie SK Shinjo SK Oba-Shinjo SM da Silva R Barbosa KC Yamamoto F. Methylenetetrahydrofolate reductase gene polymorphism is not related to the risk of ischemic cerebrovascular disease in a Brazilian population. Clinics. (2007) 62:295–300. 10.1590/S1807-59322007000300014 17589670

[31] Pramukarso DT Faradz SM Sari S Hadisaputro S. Association between methylenetetrahydrofolate reductase (MTHFR) polymorphism and carotid intima medial thickness progression in post ischaemic stroke patient. Ann Transl Med. (2015) 3:324. 10.3978/j.issn.2305-5839.2015.12.22 26734634 PMC4691009

[32] Shi Y Zhou W Cheng M Yu C Wang T Zhu L. Association of plasma bilirubin levels with peripheral arterial disease in chinese hypertensive patients: new insight on sex differences. Front Physiol. (2022) 13:867418. 10.3389/fphys.2022.867418 35492585 PMC9047868

[33] Zeng Y Li FF Yuan SQ Tang HK Zhou JH He QY. Prevalence of hyperhomocysteinemia in China: an updated meta-analysis. Biology. (2021) 10:959. 10.3390/biology10100959 34681058 PMC8533293

[34] Lupi-Herrera E Soto-López ME Lugo-Dimas AJ Núñez-Martínez ME Gamboa R Huesca-Gómez C. Polymorphisms C677T and A1298C of MTHFR gene: homocysteine levels and prothrombotic biomarkers in coronary and pulmonary thromboembolic disease. Clin Appl Thromb Hemost. (2019) 25:1076029618780344. 10.1177/1076029618780344 29916259 PMC6714945

[35] Mao Y Yang L Chen Q Li G Sun Y Wu J. The influence of CYP1A1 and CYP1A2 polymorphisms on stroke risk in the Chinese population. Lipids Health Dis. (2020) 19:221. 10.1186/s12944-020-01370-z 33046100 PMC7552501

[36] Alhazzani AA Mahfouz AA Abolyazid AY Awadalla NJ. Risk factors of the first-time stroke in the southwest of Saudi Arabia: a case-control study. Brain Sci. (2021) 11:222. 10.3390/brainsci11020222 33670278 PMC7918495

[37] Limardi PC Oktavianthi S Priliani L Lestari R Saraswati MR Suastika K. Transcription factor 7-like 2 single nucleotide polymorphisms rs290487 and rs290481 are associated with dyslipidemia in the Balinese population. PeerJ. (2022) 10:e13149. 10.7717/peerj.13149 35341056 PMC8953500

[38] Wang J Xu L Xia H Li Y Tang S. Association of MTHFR C677T gene polymorphism with metabolic syndrome in a Chinese population: a case-control study. J Int Med Res. (2018) 46:2658–69. 10.1177/0300060518768969 29658358 PMC6124264

[39] Jiang S Zhao R Pan M Venners SA Zhong G Hsu YH. Associations of MTHFR and MTRR polymorphisms with serum lipid levels in Chinese hypertensive patients. Clin Appl Thromb Hemost. (2014) 20:400–10. 10.1177/1076029612467226 23188888

[40] Yilmaz H Agachan B Isbir T Akoglu E. Is there additional effect of MTHFR C677T mutation on lipid abnormalities in renal allograft recipients? Transplant Proc. (2003) 35:1390–2. 10.1016/S0041-1345(03)00454-8 12826168

[41] Zhang L Yin RX Liu WY Miao L Wu DF Aung LH. Association of methylenetetrahydrofolate reductase C677T polymorphism and serum lipid levels in the Guangxi Bai Ku Yao and Han populations. Lipids Health Dis. (2010) 9:123. 10.1186/1476-511X-9-123 20977771 PMC2987990

[42] Chen CH Chen PY Chen CY Chiu CC Lu ML Huang MC. Associations of genetic variants of methylenetetrahydrofolate reductase and serum folate levels with metabolic parameters in patients with schizophrenia. Int J Environ Res Public Health. (2021) 18:11333. 10.3390/ijerph182111333 34769853 PMC8583146

[43] Liang R Zhou Y Xie J Lv W Kang B Liang Y. Association of C677T gene polymorphisms of methylenetetrahydrofolate reductase and plasma homocysteine level with hyperlipidemia. Nan Fang Yi Ke Da Xue Xue Bao. (2014) 34:1195–8. 25176095

[44] Werstuck GH Lentz SR Dayal S Hossain GS Sood SK Shi YY. Homocysteine-induced endoplasmic reticulum stress causes dysregulation of the cholesterol and triglyceride biosynthetic pathways. J Clin Invest. (2001) 107:1263–73. 10.1172/JCI11596 11375416 PMC209295

[45] Velez-Carrasco W Merkel M Twiss CO Smith JD. Dietary methionine effects on plasma homocysteine and HDL metabolism in mice. J Nutr Biochem. (2008) 19:362–70. 10.1016/j.jnutbio.2007.05.005 17707632 PMC2430472

[46] Liang Z Li W Yang S Liu Z Sun X Gao X. Tangier disease may cause early onset of atherosclerotic cerebral infarction: a case report. Medicine. (2018) 97:e12472. 10.1097/MD.0000000000012472 30278532 PMC6181625

[47] Ma W Ren X Zhang L Dong H Lu X Feng W. Apolipoprotein E gene polymorphism and coronary artery disease risk among patients in Northwest China. Pharmgenomics Pers Med. (2021) 14:1591–9. 10.2147/PGPM.S338285 34908864 PMC8665779

[48] Ljunggren S Bengtsson T Karlsson H Starkhammar Johansson C Palm E Nayeri F. Modified lipoproteins in periodontitis: a link to cardiovascular disease? Biosci Rep. (2019) 39:BSR20181665. 10.1042/BSR20181665 30842338 PMC6434390

